# Dihydrodinophysistoxin-1 Produced by *Dinophysis norvegica* in the Gulf of Maine, USA and Its Accumulation in Shellfish

**DOI:** 10.3390/toxins12090533

**Published:** 2020-08-20

**Authors:** Jonathan R. Deeds, Whitney L. Stutts, Mary Dawn Celiz, Jill MacLeod, Amy E. Hamilton, Bryant J. Lewis, David W. Miller, Kohl Kanwit, Juliette L. Smith, David M. Kulis, Pearse McCarron, Carlton D. Rauschenberg, Craig A. Burnell, Stephen D. Archer, Jerry Borchert, Shelley K. Lankford

**Affiliations:** 1 Office of Regulatory Science, United States Food and Drug Administration, Center for Food Safety and Applied Nutrition, College Park, MD 20740, USA; wlstutts@ncsu.edu (W.L.S.); MaryDawn.Celiz@fda.hhs.gov (M.D.C.); 2 Maine Department of Marine Resources, West Boothbay Harbor, ME 05475, USA; Jill.MacLeod@maine.gov (J.M.); Amy.Hamilton@maryland.gov (A.E.H.); Bryant.J.Lewis@maine.gov (B.J.L.); David.W.Miller@maine.gov (D.W.M.); Kohl.Kanwit@maine.gov (K.K.); 3 Virginia Institute of Marine Science, College of William and Mary, Gloucester Point, VA 23062, USA; jlsmith@vims.edu; 4 Department of Biology, Woods Hole Oceanographic Institute, Woods Hole, MA 02543, USA; dkulis@whoi.edu; 5 Biotoxin Metrology, National Research Council Canada, Halifax, NS B3H 3Z1, Canada; Pearse.McCarron@nrc-cnrc.gc.ca; 6 Bigelow Analytical Services, Bigelow Laboratory for Ocean Sciences, East Boothbay, ME 04544, USA; carlton.rauschenberg@gmail.com (C.D.R.); cburnell@bigelow.org (C.A.B.); sarcher@bigelow.org (S.D.A.); 7 Washington State Department of Health, Olympia, WA 98504, USA; Jerry.Borchert@DOH.WA.GOV; 8 Washington State Department of Health Public Health Laboratories, Shoreline, WA 98155, USA; Shelley.Lankford@DOH.WA.GOV

**Keywords:** diarrhetic shellfish poisoning, dihydro-DTX1, *Dinophysis norvegica*, Gulf of Maine USA

## Abstract

Dihydrodinophysistoxin-1 (dihydro-DTX1, (M-H)^−^ *m/z* 819.5), described previously from a marine sponge but never identified as to its biological source or described in shellfish, was detected in multiple species of commercial shellfish collected from the central coast of the Gulf of Maine, USA in 2016 and in 2018 during blooms of the dinoflagellate *Dinophysis norvegica*. Toxin screening by protein phosphatase inhibition (PPIA) first detected the presence of diarrhetic shellfish poisoning-like bioactivity; however, confirmatory analysis using liquid chromatography-tandem mass spectrometry (LC-MS/MS) failed to detect okadaic acid (OA, (M-H)^−^ *m/z* 803.5), dinophysistoxin-1 (DTX1, (M-H)^−^ *m/z* 817.5), or dinophysistoxin-2 (DTX2, (M-H)^−^ *m/z* 803.5) in samples collected during the bloom. Bioactivity-guided fractionation followed by liquid chromatography-high resolution mass spectrometry (LC-HRMS) tentatively identified dihydro-DTX1 in the PPIA active fraction. LC-MS/MS measurements showed an absence of OA, DTX1, and DTX2, but confirmed the presence of dihydro-DTX1 in shellfish during blooms of *D. norvegica* in both years, with results correlating well with PPIA testing. Two laboratory cultures of *D. norvegica* isolated from the 2018 bloom were found to produce dihydro-DTX1 as the sole DSP toxin, confirming the source of this compound in shellfish. Estimated concentrations of dihydro-DTX1 were >0.16 ppm in multiple shellfish species (max. 1.1 ppm) during the blooms in 2016 and 2018. Assuming an equivalent potency and molar response to DTX1, the authority initiated precautionary shellfish harvesting closures in both years. To date, no illnesses have been associated with the presence of dihydro-DTX1 in shellfish in the Gulf of Maine region and studies are underway to determine the potency of this new toxin relative to the currently regulated DSP toxins in order to develop appropriate management guidance.

## 1. Introduction

Shellfish harvesting closures due to Diarrhetic Shellfish Toxins (DSTs) in excess of the 0.16 ppm total okadaic acid equivalents (OA eq.) regulatory guidance level are a relatively recent occurrence in the United States (US). The first such closure occurred in the state of Texas (Gulf of Mexico region) in 2008 due to a bloom of *Dinophysis ovum* [1,2]. The first closure in the state of Washington (west coast Puget Sound region) occurred in 2011 due to a mixture of species, primarily *D. acuminata* [3,4]. On the east coast of the US, a large bloom of *D. acuminata* prompted a precautionary shellfish harvesting closure in the Potomac River bordering the states of Maryland and Virginia in 2002, but only trace concentrations of DSTs were found [5]. More recently, *D. acuminata* has been documented in increasing abundance in the Mid-Atlantic region with DSTs in excess of guidance levels in shellfish (non-commercial) occurring sporadically since 2011 [6,7], but to date, no additional shellfish harvesting closures have occurred in this region. In the east coast New England region, limited shellfish harvesting closures have occurred in the Nauset Marsh system in Massachusetts since 2015 due to DSTs from blooms of *D. acuminata* (M Brosnahan, Woods Hole Oceanographic Institute, personal communication). 

**Figure 1:**
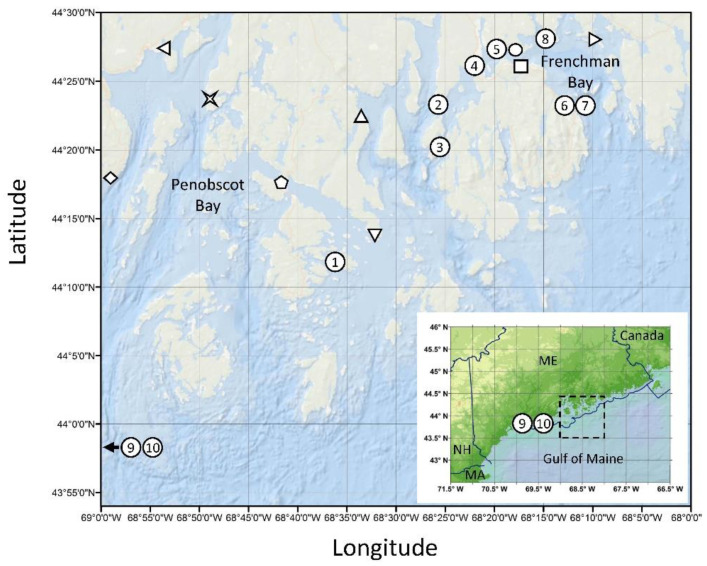
Map of sampling locations during the 2016 *Dinophysis norvegica* bloom on the central coast of the Gulf of Maine, USA. Symbols indicate sampling locations for both water and shellfish and correspond to data in Figure 2: [◇] Lincolnville, [◁] Searsport, [ 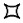] Dice Head, [⬠] Eggemoggin Reach, [△] Blue Hill Falls, [▽] Flye Point, [◯] Lamoine State Park, [☐] Salsbury Cove, [▷] Waukeag. Numbers indicate shellfish sampling only and correspond to data in Table 1: (1) Stinson Neck Causeway, (2) Oak Point, (3) Pretty Marsh Harbor, (4) Trenton Sea Plane Ramp, (5) Googins Ledge, (6,7) Bar Harbor, (8) Raccoon Cove, (9,10) Lumbo’s Hole (control site outside of bloom area). Abbreviations: ME—Maine, NH—New Hampshire, MA—Massachusetts. Sampling site coordinates provided in Table S1.

**Figure 2:**
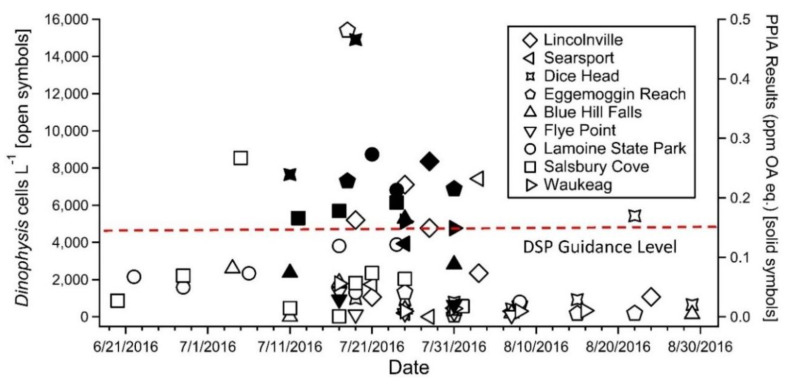
*Dinophysis* spp. cell counts (open symbols) and corresponding protein phosphatase inhibitory activity (closed symbols) in shellfish collected during the 2016 *Dinophysis norvegica* bloom in the Gulf of Maine. Dashed line indicates guidance level for DSP toxins in shellfish of 0.16 ppm okadaic acid equivalents (OA eq.). Symbols correspond to sampling sites in Figure 1. Sampling site coordinates provided in Table S1.

Although *D. acuminata* and *D. ovum* have been responsible for the majority of diarrhetic shellfish poisoning (DSP)-related shellfish harvesting closures in the US to date, other potentially toxigenic species have been documented in lower abundance, namely *D. fortii* and *D. norvegica* [4,7]. In the central coast of the Gulf of Maine, *Dinophysis* spp. commonly reach peak abundances in the summer months, and when blooms occur, they are typically predominated by *D. norvegica* (Maine Department of Marine Resources, personal communication). From July 5 to August 29, 2016, a large monospecific bloom of *D. norvegica* occurred in the Penobscot and Frenchman Bay regions of the central coast of the Gulf of Maine, USA (Figure 1). Multiple samples with cell concentrations > 2000 cells L^−1^ were observed with a maximum concentration of 54,300 cells L^−1^ recorded on July 17. Shellfish collected from several sites throughout the bloom area were screened using a commercial protein phosphatase inhibition assay (PPIA), which indicated the presence of DSTs in excess of 0.16 ppm, prompting a ban on shellfish harvesting on July 20 (Figure 2). Subsequent confirmatory testing using liquid chromatography tandem mass spectrometry (LC-MS/MS) failed to detect OA, dinophysistoxin-1 (DTX1), or dinophysistoxin-2 (DTX2) from samples collected during the bloom. Testing in three additional laboratories using both methods provided the same results, positive by PPIA and negative by LC-MS/MS. Further exploratory testing using liquid chromatography-high resolution mass spectrometry (LC-HRMS) to screen for additional lipophilic shellfish toxins was also negative. Therefore, the harvesting ban was lifted on August 5 and the PPIA results were considered as false positives. 

In 2017, an investigation was initiated to determine the source of the DST-like bioactivity occurring in shellfish harvested during blooms of *D. norvegica* in Maine. Further testing using several commercial enzyme-linked immuno-sorbent assays (ELISAs) specific for DSTs provided the same results as those found by PPIA testing, confirming the presence of compound(s) with both structures and bioactivities similar to DSTs. Bioactivity-guided fractionation identified a single PPIA-positive fraction similar in retention time to DTX1. LC-MS/MS analyses of this semi-purified fraction tentatively identified the unknown DST as dihydro-DTX1. This same compound was identified in additional shellfish samples collected during the *D. norvegica* bloom in 2016 and again in 2018, as well as in a filtered plankton sample collected in 2018. Finally, this compound was confirmed to be the only DST produced in two cultured isolates of *D. norvegica* collected from the Gulf of Maine in 2018. Detailed here are the investigations leading to the discovery of this novel DST as well as a proposed strategy to manage this potential new DSP risk until an analytical standard can be produced and the potency of this new toxin in relation to the other known DSTs can be determined.

## 2. Results

### 2.1. Initial Testing of Shellfish Collected during the 2016 D. norvegica Bloom in the Gulf of Maine

The 2016 *D. norvegica* bloom in the Penobscot and Frenchman Bay regions of the central coast of the Gulf of Maine lasted for approximately two months (July 5th–August 29th) (Figure 1 and Figure 2). During that time, numerous shellfish samples were found to display DST-like activity, based on PPIA screening, in excess of 0.16 ppm (Figure 2), but subsequent testing for OA, DTX1, and DTX2 by confirmatory LC-MS/MS analysis could not confirm the presence of these compounds.

In an attempt to resolve this discrepancy, ten initial samples of frozen homogenized mussels (*Mytilus edulis*) were sent to three additional laboratories for follow-up testing. Eight were samples collected during the 2016 *D. norvegica* bloom that had all previously tested positive for DST-like activity based on PPIA screening while an additional two samples, collected prior to the bloom and outside of the bloom area, were sent as negative controls. With the exception of one sample, all samples were found to be <LOD for OA, DTX1, and DTX2 by LC-MS/MS testing. All samples were also shown to be <LOD for the additional lipophilic shellfish toxins azaspiracids, pectenotoxins, and yessotoxins as tested using LC-HRMS analysis. The single sample found to contain a detectable concentration of DSTs by LC-MS/MS was actually one of the presumptive negative control samples collected on 6/27/16, prior to the *D. norvegica* bloom and from south of the bloom area (Sample 10 from Figure 1; 0.04 and 0.05 ppm DTX1 only as tested by two independent laboratories). All samples were re-tested at the FDA Center for Food Safety and Applied Nutrition (CFSAN) by PPIA and LC-MS/MS and results were found to be consistent with previous testing. Further testing using both a qualitative lateral flow ELISA (NEOGEN Reveal 2.0 for DSP) and a quantitative ELISA (Bioo Scientific MaxSignal Okadaic Acid (DSP)) showed results consistent with PPIA testing, suggesting the presence of DST-like compound(s) based on both bioactivity and structure, but a compound distinct from OA, DTX1, DTX2, or any esterified derivatives thereof (Table 1).

### 2.2. Bioactivity-Guided Fractionation

**Figure 3:**
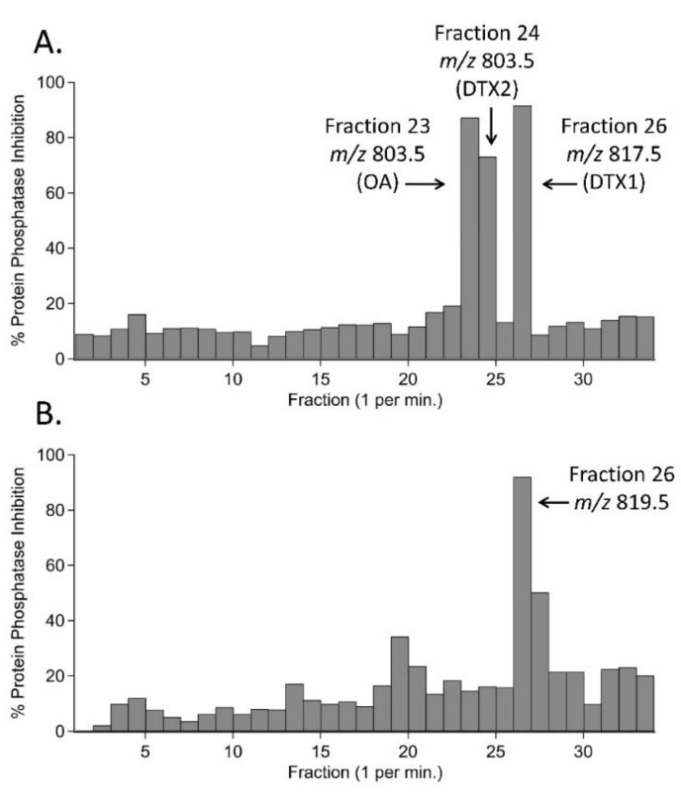
Results from the bioactivity-guided fractionation procedure for (**A**) control extract of clam (*Mercenaria mercenaria*) homogenate spiked with 0.16 ppm each of OA, DTX1, and DTX2, and (**B**) extract of mussel (*Mytilus edulis*) collected during 2016 *Dinophysis norvegica* bloom in the Gulf of Maine (corresponds to Sample 2 in Figure 1 and Table 1). LC-MS/MS confirmed OA, DTX2, and DTX1 in the spiked control sample, and the tentative toxin at (M-H)^−^ *m/z* 819.5 in the mussel extract.

To first test the utility of the bioactivity-guided fractionation procedure, 500 µL of hydrolyzed extract of clam (*Mercenaria mercenaria*) homogenate that had previously been spiked with 0.16 ppm each of OA, DTX1, and DTX2 was fractionated and screened for PPIA activity. Three fractions were found to contain DST-like activity: the fractions collected from 23–24 min, 24–25 min, and 26–27 min. Subsequent testing of these fractions by LC-MS/MS confirmed them as containing OA, DTX2, and DTX1, respectively (Figure 3A). Next, 500 µL of hydrolyzed mussel extract that showed the highest PPIA activity from the initial set of 10 samples supplied by ME Department of Marine Resources (DMR) (Sample 2 from Table 1, >0.35 ppm OA eq. activity using the PPIA kit and 0.67 ppm OA eq. using the MaxSignal quantitative ELISA) was also fractionated. PPIA testing showed a prominent peak in bioactivity in the 26-27 min fraction, which was closest in elution order to DTX1 (Figure 3B). To determine the molecular ion of the compound responsible for the PPIA activity in fraction 26 of the mussel extract, full-scan experiments were performed by Q1 scanning in negative polarity (see Section 4.5.2). From an extracted ion chromatogram for *m/z* 800–840, the expected mass range for DST-like compounds, an abundant peak was observed at a retention time of 7.3 min, close to the known retention time of DTX1, using the chromatographic method described herein. The predominant ion observed in negative ionization mode for this chromatographic peak was *m/z* 819.5, two Da larger than DTX1.

A product ion scan was then performed to obtain MS/MS fragmentation information for *m/z* 819.5 and to select product ions for SRM method development. The top three most abundant product ions were the same as those for known DSTs, *m/z* 255.2, 151.1, and 131.1 (Figure S1).

### 2.3. Liquid Chromatography-High Resolution Mass Spectrometry (LC-HRMS) Measurements of the PPIA Active Fraction

**Figure 4:**
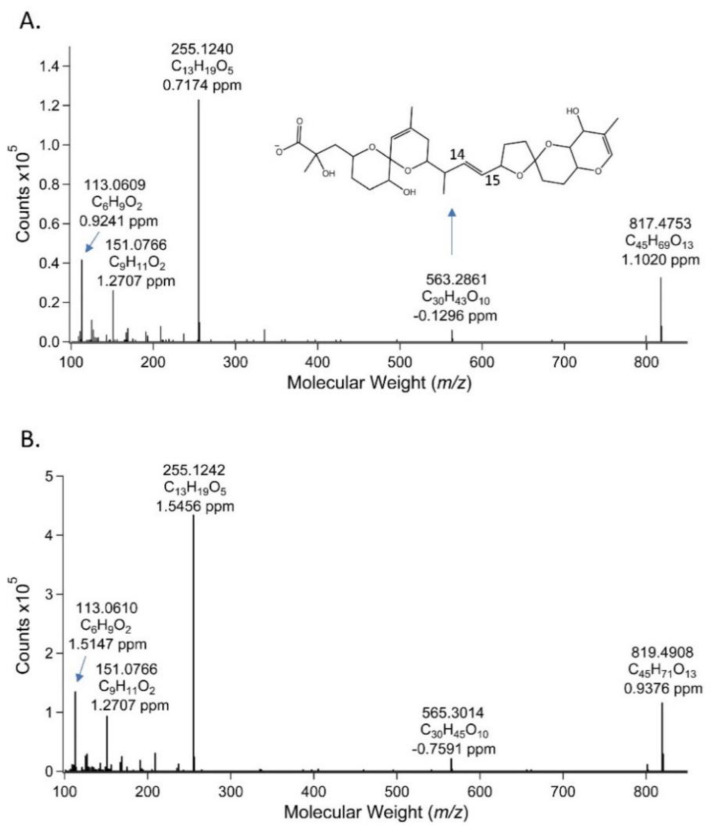
MS/MS spectra of (**A**) DTX1 certified reference material at (M-H)^−^ *m/z* 817.5 and (**B**) suspect DST at (M-H)^−^ *m/z* 819.5 from the 26-27-minute fraction from the PPIA bioactivity-guided fractionation procedure (depicted in Figure 3) for Sample 2 from Figure 1 and Table 1. Structure in panel A depicts (M-H)^−^ *m/z* 563.2861 product ion showing the location of the 14-15 double bond. Structure of corresponding product ion in panel B with (M-H)^−^ *m/z* 565.3014, from compound tentatively identified as dihydro-DTX1, has yet to be confirmed. Full structures of OA, DTX1, and DTX2, as well as proposed structures for the 255.2, 151.1, and 113.1 product ions used in the SRM analysis, are provided in Figure S2.

A peak detected in the 26-27 min fraction showed a measured accurate mass of *m/z* 819.4908, which corresponds to the (M-H)^−^ of C_45_H_71_O_13_ (∆m = 0.9376 ppm). This compound differed from the measured accurate mass for a DTX1 standard of *m/z* 817.4753 (∆m = 1.1020 ppm for C_45_H_69_O_13_) by the addition of two hydrogen atoms, suggesting it was a dihydro derivative of DTX1. Furthermore, the LC-high-resolution MS/MS spectrum of this compound showed several product ions that were also consistent with the MS/MS spectrum of the DTX1 standard. These product ions were *m/z* 255.1240 as the most abundant ion, followed by *m/z* 113.0609 and *m/z* 151.0766. The corresponding product ions in the DTX1 standard were *m/z* 255.1242, *m/z* 113.0610, and *m/z* 151.0766. An additional product ion observed for the tentative dihydro-DTX1 was *m/z* 565.3014, corresponding to the chemical formula C_30_H_45_O_10_ (∆m = −0.7591 ppm), and is 2 mass units higher than the corresponding product ion (*m/z* 563.2861; C_30_H_43_O_10,_ ∆m = −0.1296 ppm) for DTX1, indicating that the two additional hydrogen atoms are contained in this segment of the molecule (Figure 4). Specifically, the double bond at C14,15 is potentially modified on the tentative dihydro-DTX1.

### 2.4. Selected Reaction Monitoring (SRM) Analysis for DSP Toxins and Dihydro-DTX1 in Water and Shellfish Samples and Comparison with PPIA

**Figure 5:**
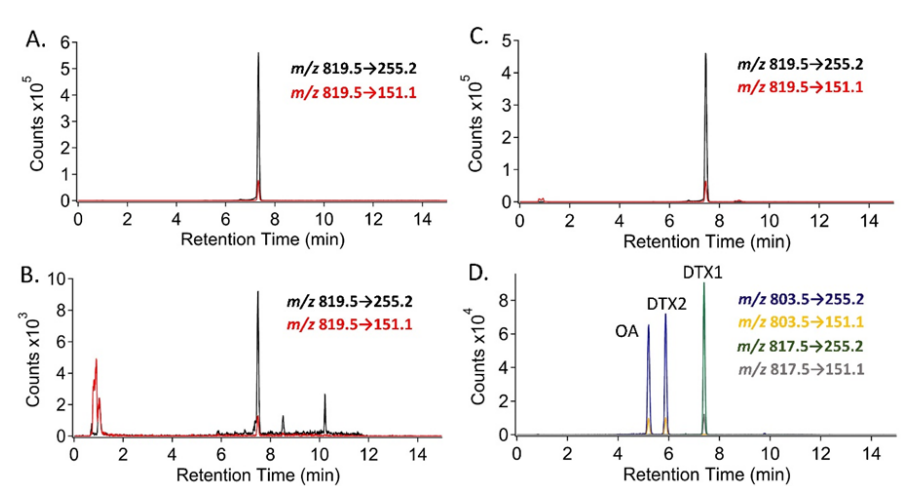
Extracted ion chromatograms from LC-MS/MS SRM analysis for dihydro-DTX1 ((M-H)^−^ *m/z* 819.5), fragmenting to *m/z* 255.2 (for quantitation) (black trace) and 151.1 (for confirmation) (red trace), for (**A**) 26–27 min fraction from bioactivity guided fractionation procedure on mussel (*M. edulis*) extract from 2016 *D. norvegica* bloom in the Gulf of Maine, (**B**) filtered water sample collected during 2018 *D. norvegica* bloom in the Gulf of Maine, and (**C**) representative mussel (*M. edulis*) sample collected during 2018 *D. norvegica* bloom in the Gulf of Maine. OA ((M-H)^−^ *m/z* 803.5), DTX1 ((M-H)^−^ *m/z* 817.5), and DTX2 ((M-H)^−^ *m/z* 803.5), all fragmenting to *m/z* 255.2 and 151.1, were monitored for and were not detected (not shown). Panel (**D**) shows extracted ion chromatograms for a 12.8 ng/mL standard mix of OA, DTX1, and DTX2 run in the same analytical batch as panel C for comparison of relative retention times using the current chromatography for the approved LC-MS/MS method.

Using the data acquired from the previous analyses, minor modifications were made to the NSSP-approved LC-MS/MS method for the analysis of total (free plus esterified) DSP toxins in shellfish to include the preliminary detection of dihydro-DTX1 (detailed in Section 4.5.4 below). Using the modified LC-MS/MS method with data acquired in SRM scan mode, samples collected from the Gulf of Maine in 2016 and 2018 during blooms of *D. norvegica* were analyzed for the presence of these four compounds, and dihydro-DTX1 was confirmed to be the only DST present in shellfish as well as water (Figure 5).

**Figure 6:**
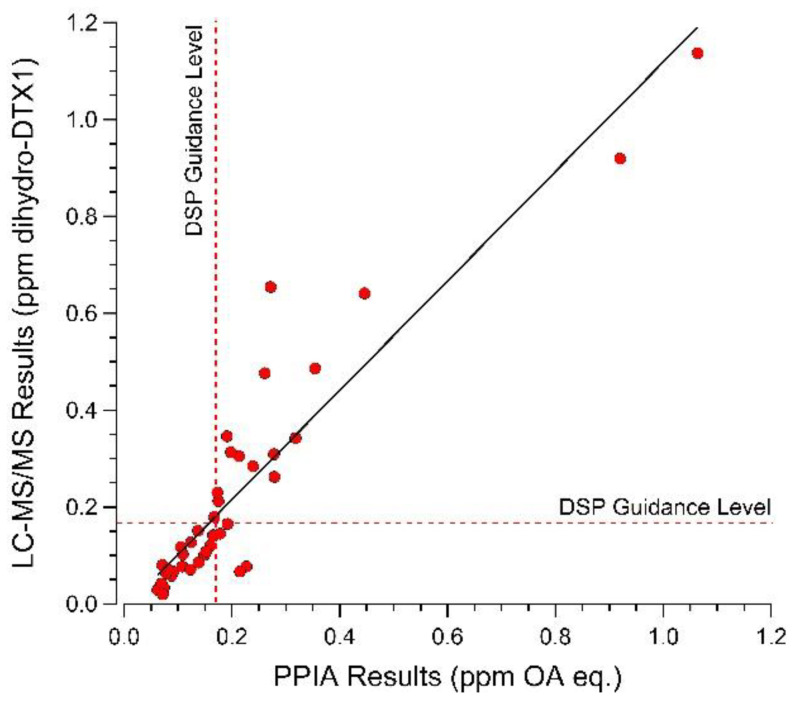
Comparison of protein phosphatase inhibition, as measured using a commercial PPIA kit, and dihydro-DTX1 concentration, as measured by LC-MS/MS SRM analysis, for mussels (*Mytilus edulis*), oysters (*Crassostrea virginica*), and clams (*Spisula solidissima,* and *Mya arenaria*) (N = 42 total) collected in 2016 and 2018 from the central coast of the Gulf of Maine during blooms of *Dinophysis norvegica*. Dashed line indicates DSP guidance level of 0.16 ppm OA eq. Solid line indicates best fit linear regression for the two data sets (slope 1.13 ± 0.07, r^2^ 0.86).

Next, the original 10 shellfish samples analyzed by multiple labs in 2016 were re-analyzed using the modified LC-MS/MS method and samples 1–8, collected during the 2016 *D. norvegica* bloom, were all confirmed to contain dihydro-DTX1 at concentrations that matched well with previous results generated by PPIA and ELISA (Table 1). In addition, shellfish samples from multiple species, including mussels (*Mytilus edulis*), oysters (*Crassostrea virginica*), and clams (*Spisula solidissima* and *Mya arenaria*), collected in 2016 and 2018 (N = 48 total) were analyzed both by PPIA and by the modified LC-MS/MS method and results were compared using linear regression and correlation analyses for any sample >LOD of the PPIA kit (N = 42). In the absence of an analytical standard for dihydro-DTX1, a DTX1 standard was used for external calibration, assuming an equivalent molar response [8]. The linear regression analysis found a slope of 1.13 ± 0.07, with an r^2^ of 0.86. Correlation analysis of results from these same samples found the two analyses to be significantly correlated (*p* < 0.0001) with a Pearson r score of 0.9260 (Figure 6).

**Figure 7:**
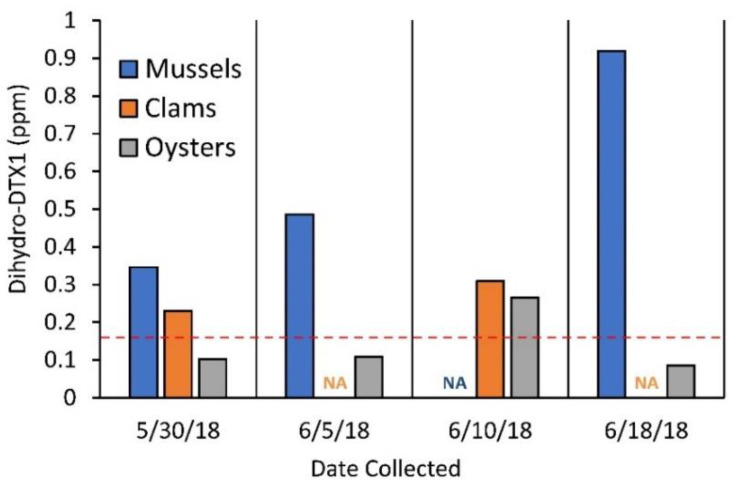
Dihydro-DTX1 accumulation in mussels (*Mytilus edulis*), clams (*Spisula solidissima*), and oysters (*Crassostrea virginica*) collected between May 30th and June 18th from Blue Hill Falls, Maine during the 2018 Gulf of Maine *Dinophysis norvegica* bloom. Each bar represents a single composite shellfish sample (≥12 individuals each). Dashed line indicates DSP guidance level of 0.16 ppm OA eq.

Lastly, in order to test for species-specific differences in the bioaccumulation of dihydro-DTX1 in various commercial shellfish species from the Gulf of Maine, three species of shellfish: mussels (*M. edulis*), clams (*S. solidissima*), and oysters (*C. virginica*), were collected approximately weekly between May 30th and June 18th from a single location (Blue Hill Falls, symbol (△) from Figure 1) during the 2018 *D. norvegica* bloom and analyzed by LC-MS/MS. All three species were found to bioaccumulate dihydro-DTX1 with the rank order mussels > clams > oysters. Even though clams and oysters were found to accumulate less toxin overall compared to mussels, all three species would have exceeded regulatory guidance levels on at least one occasion during the course of the bloom, assuming equivalent potency and molar response to DTX1 (Figure 7).

### 2.5. Production of Dihydro-DTX1 in a Culture of Gulf of Maine D. norvegica

Isolates of *D. norvegica* from the Gulf of Maine contained dihydro-DTX1 and pectenotoxin 2 (PTX2) in both the intracellular and extracellular fractions of the cultures (Table 2). OA, DTX1, and DTX2 were not detected in the extracts of *D. norvegica* cultures. 

Once subjected to alkaline hydrolysis, the toxin concentrations of dihydro-DTX1 in the intracellular fraction increased 60–80 fold, indicating the extensive presence of esterified toxin derivatives (Table 2). More specifically, the esterified toxins made up 98% of the total intracellular dihydro-DTX1 pool (free + esterified dihydro-DTX1) in both isolates. Extracellular dihydro-DTX1 derivatives were also present in the medium and dominated over the parent toxin with 80–83% present as esterified toxins. Overall, the amount of the parent congener, dihydro-DTX1, was similar inside and outside the cells; however, once esterified dihydro-DTX1 toxins were included in the analysis, significantly more toxin was located within the cells.

## 3. Discussion

Multiple cases of severe vomiting and diarrhea associated with the consumption of mussels and scallops, with a failure to detect any known pathogenic microorganisms, occurring in Japan in 1976–1977 led to the first description of the syndrome known as Diarrhetic Shellfish Poisoning (DSP) [9]. The source organism for this toxic syndrome, in Japan, was determined to be the dinoflagellate *Dinophysis fortii* and the unknown toxin was named dinophysistoxin [10]. Structural elucidation of this toxin, termed dinophysistoxin-1 (DTX1), determined it to be a novel polyether derivative of a C38 fatty acid that was closely related to the similar toxins okadaic acid (35-desmethyl-DTX1) and acanthifolicin (9,10-episulfide-35-desmethyl-DTX1) that had previously been identified during bioassay-guided searches for novel antitumor agents from marine sponges [11,12,13]. OA was later determined to also be produced by dinoflagellates, such as *Prorocentrum lima* and *D. acuminata* and to be the primary DSP toxin found in shellfish in Europe [14,15,16]. Another dinophysistoxin (DTX2, 31-desmethyl-35-methyl-OA) was later found in shellfish from Ireland [17]. To date, these three toxins, along with their 7-*O*-acyl shellfish-derived metabolites, have been the primary toxins found in shellfish associated with DSP events worldwide.

Since the original descriptions of OA, DTX1, and DTX2, a limited number of additional DST-derivatives have been described, although they have never been found to be present in shellfish in sufficient quantities to cause DSP. 2-Deoxy-OA and 7-deoxy-OA were both isolated as minor constituents from the dinoflagellate *P. lima* [18,19], 19-epi-OA was isolated as a minor constituent from *P. belezianum* [20], 19-epi-DTX1 and 19-epi-DTX2 were identified as minor impurities during the production of DSP-certified reference materials [21], while 14,15-dihydro-OA was isolated as a minor constituent from the marine sponge *Halichondria okadai* during the original isolation of OA [22]. Using a similar bioassay-guided isolation approach as was used to first identify OA from *H. okadai*, with the goal of identifying novel biologically active compounds from marine organisms, 14,15-dihydrodinophysistoxin-1, along with roughly equivalent amounts of OA and DTX1, was isolated from marine sponges (*Phakellia* sp.) collected in waters from the central coast of Maine in 1985 and 1986 [18,23]. Although this compound was first reported over 20 years ago, its biological source was never determined, and it has not appeared in the scientific literature again to date. 

The first record of *D. norvegica* blooming in North America occurred in Bedford Basin, Nova Scotia on the Atlantic coast of Canada in 1990 [24]. Maximum cell densities were 4.5 × 10^5^ cells L^−1^ and DST(s) were reported to be present both in net tow plankton samples as well as in experimentally exposed scallops (*Placopecten magellanicus*) based on a commercial ELISA. In that same year, the first cases of DSP were reported in North America from mussels harvested from Mahone Bay, Nova Scotia, Canada [25,26]. During that event, *D. norvegica* was reported in plankton samples collected from Mahone Bay, but no DSP toxins were detected [25] (methods not described). Shellfish from the DSP event were positive for DST-like activity by mouse bioassay and DTX1 was confirmed by a combination of methods including LC-MS and proton NMR spectroscopy [26]. *P. lima*, cultured from water samples collected during the same time period, was subsequently shown to produce both OA and DTX1 and the event was attributed to *P. lima* [25]. At that time, the methods required to culture *Dinophysis* spp. had not yet been established, so the contribution of *Dinophysis* to this DSP event could not be determined. 

Not long after the first DSP event in eastern Canada, several unexplained incidents of shellfish-related gastroenteritis in Maine, USA prompted a study to assess the prevalence of potential DST-producing organisms and the occurrence of DST-like toxicity in mussels along the entire state coast [27]. In that study, DST-like activity in mussel hepatopancreas was detected using a PPIA test only in the central coastal region in the vicinity of Eastern Bay and Frenchman Bay, the same region as our current study. The most prevalent potentially DST-producing species found in this region at the time was *D. norvegica*, but analysis of net tow samples were negative for protein phosphatase inhibitory (PPI) activity. Both mussels and plankton were also negative for OA and DTX1 by LC-MS/MS testing, but the authors noted that the bioactivity detected in mussels by PPIA was likely below the detection limit for LC-MS/MS. Although lower in number, *P. lima* was documented in the epiphytic community and testing of a concentrated sample of this material detected DST-like activity using the PPIA test with the presence of DTX1 confirmed by LC-MS/MS; therefore, the authors concluded that *P. lima* was the source of the PPI activity in mussels during that study. Follow-on studies performed between 2001 and 2003 looking at the seasonal distribution of potential DST-producing dinoflagellates and DSP-like bioactivity in plankton and shellfish in several northeastern states found a weak but significant correlation between PPI activity and the presence of *P. lima* in the epibiota [28,29]. The authors also noted the presence of both *D. acuminata* and *D. norvegica* throughout the study region, with *D. norvegica* being the more prevalent of the two species in the coastal Gulf of Maine, but found PPI activity associated with a bloom of *D. norvegica* only once [29]. Overall, toxins in shellfish were found only rarely and only at low levels and therefore the authors concluded that the threat of DSP in the region was minimal despite the presence of several potential DSP-producing species [29]. 

We show here that *D. norvegica* in the central coast of the Gulf of Maine, both in culture and in-situ, produces a DST-like toxin distinct from OA, DTX1, or DTX2, the only DSTs associated with DSP worldwide to date. The toxin has been tentatively identified as a dihydro-derivative of DTX1 and is likely 14,15 dihydro-DTX1 as was described from marine sponges collected from this same region over 30 years ago, although confirmation of this will require further structural elucidation studies. The presence of this toxin, as quantified against a DTX1 standard by LC-MS/MS analysis, the current reference method for DST testing under the National Shellfish Sanitation Program in the US [30], correlated well with PPIA testing, indicating that this toxin alone can explain the DST-like activity detected in this region in association with blooms of *D. norvegica* in 2016 and 2018. This also suggests that dihydro-DTX1 binds equivalently to the PP2A enzyme used in the commercial PPIA kit compared to OA, the standard utilized in the kit. During the original description of 14,15-dihydro-DTX1, this compound was reported to be equally potent as compared to OA and DTX1 using a cytotoxicity assay against L-1210 leukemia cells [23], but in other studies looking at the relative inhibitory potencies of various OA-derivatives, including 14,15-dihydro-OA, this compound was reported to have a higher dissociation constant (lower affinity) for both PP1 and PP2A, isolated from rabbit skeletal muscle, compared to OA and DTX1 [22]. This was proposed to be due to the conversion of the double bond between C-14 and C-15 to a single bond, which the authors hypothesized to stabilize the circular confirmation known to be important for the binding of these compounds to protein phosphatases [22]. Determination of the relative potency of the dihydro-DTX1 produced by *D. norvegica* compared to the other known DSP-causing DSTs will require further testing once a better characterized, analytically pure preparation of this compound is available. In the meantime, it appears that PPIA screening with confirmatory LC-MS/MS testing utilizing *m/z* 819.5 with product ions at *m/z* 255.2, for quantitation, and 151.1 and/or 113.1 for confirmation, in negative ion mode with acidic chromatography according to the current NSSP method is a viable means of screening for this toxin in shellfish until a formal analyte extension study can be performed. As with all marine biotoxins, chemical analytical methods such as HPLC or LC-MS/MS depend greatly on the availability of accurate toxicity equivalency factors (TEFs) [31]. The determination of a TEF for the dihydro-DTX1 compound is a priority so that the risk this toxin poses to human consumers can be properly assessed, especially considering that this toxin has not been conclusively linked to any DSP-like illnesses to date. This work is currently in progress. 

The central coast of the Gulf of Maine is not the only region in the US where *D. norvegica* occurs, although it does appear to be the only region in the US that we are aware of where it is the predominant species of *Dinophysis* present. *D. norvegica* has been documented in lower abundance compared to species such as *D. acuminata* in the mid-Atlantic region [7], the Pacific Northwest [4], and along the central coast of California [32]. Historically, *D. norvegica* was shown to reach high abundances on the Atlantic coast of Canada, particularly Nova Scotia [24], and still occurs occasionally in high abundance along with *D. acuminata* in that region today (Nancy Lewis, personal communication). Worldwide, *D. norvegica* occurs in many cold-water coastal environments, such as Norway, where it commonly co-occurs with other *Dinophysis* species such as *D. acuta* and *D. acuminata* [33], and the Baltic Sea [34], but it is often considered a minor contributor to DSP events [35,36]. Studies looking at toxins in pooled cells of *D. norvegica* picked from environmental samples in Norway [37] and Japan [38] found PTX in 10/10 samples, but found low levels of OA, as measured by targeted LC-MS/MS, in only 1/10 samples, with no DTX1 or DTX2 detected. It remains to be determined if *D. norvegica* in any of these additional locations also produces the dihydro-DTX1 compound and what potential risk this poses to shellfish consumers. 

## 4. Materials and Methods

### 4.1. Phytoplankton and Shellfish Sampling 

Individual phytoplankton samples were each comprised of 10 liters of surface seawater collected from shore and gravity filtered through a 20 µm sieve. Once the seawater had fully drained, the cylinder was inverted and placed onto a funnel attached to a 50 mL centrifuge tube. Phytoplankton collected on the sieve were rinsed into the collection tube using freshly filtered seawater. Sample tubes were suspended by a rack in a cooler held between 0–10 °C during transport. Once transported to the lab, samples were held at 0–4 °C and analyzed within 24 h. If samples could not be analyzed within this timeframe, they were fixed with 80 µL of Lugol’s iodine and stored at 0–4 °C until analysis.

Swift M10 compound microscopes (Swift Optical Instruments, Schertz, TX, USA) were used at 100X magnification for phytoplankton enumeration. Each sample tube was gently inverted three times and 1 mL of the sample aliquoted onto a Sedgwick Rafter gridded slide for enumeration. Using light microscopy, 0.2 mL (200 grids) were analyzed, after which the sample was discarded, the slide rinsed with deionized water, and a second sample was loaded and analyzed. The genus *Dinophysis* was enumerated using morphological characteristics. The total counts of individual cells in the two separate aliquots were averaged with numbers reported in cells liter^−1^.

Each bivalve sample was comprised of 12–15 specimens for a given species that were composited for analysis. Samples were transported live in a cooler held at a temperature of 0–10 °C. Upon delivery to the lab, samples were stored at 0–4 °C and were processed within 24 h of collection. For processing, each sample was rinsed, drained, shucked, and the tissues were homogenized by a blender. Homogenized samples were stored at 0–4 °C and extracted within 24 h. Samples not scheduled for analysis within 24 h were frozen at −20 °C until extraction. Remaining homogenates were stored at −20 °C and after 2 months, archived in long-term storage at −80 °C. Additional aliquots for further chemical analysis as part of this study were taken from these archived samples.

### 4.2. Standards and Reagents

Certified reference materials (CRMs) for OA, DTX1, and DTX2 were purchased from the National Research Council Canada (Halifax, NS, Canada). All reagents were purchased from Sigma-Aldrich (St. Louis, MO, USA) and were of analytical grade or better. All solvents were purchased from Fisher Scientific (Pittsburgh, PA, USA) and were LC-MS grade.

### 4.3. Commercial Test Kits

Three commercial test kits for the determination of DSP toxins in shellfish were utilized in the study: 1. Okadaic Acid (DSP) PP2A kit (PN 520025, Abraxis, Inc. Warminster, PA, USA), 2. Reveal 2.0 for DSP (NEOGEN Corporation, Lansing, MI, USA), and 3. MaxSignal Okadaic Acid (DSP) ELISA Test Kit (Bioo Scientific Corp, Austin, TX, USA). The Okadaic Acid (DSP) PP2A kit is an *in-vitro* test based on the inhibition of protein phosphatase 2A, a known biological target for DSTs. The Reveal 2.0 for DSP kit is a qualitative antibody-based lateral flow test, while the MaxSignal Okadaic Acid (DSP) ELISA kit is a quantitative micro-well plate antibody-based test. For all kits, the manufacturer’s instructions were followed, with the exception of the Okadaic Acid (DSP) PP2A kit where 2 g of shellfish homogenate was extracted in a total of 20 mL solvent (1:10 dilution) as opposed to the current manufacturer’s instructions of 5 g homogenate extracted in 25 mL solvent (1:5 dilution). To compensate for the different extraction dilutions, the total volume of the dilution in buffer after sample hydrolysis was changed from 20 mL to 10 mL (Section D, Step 12 of manufacturer’s instructions). This adjustment corresponds to the standard extraction for the validated LC-MS/MS method for DSTs and allows the same extract to be used for both analyses. 

### 4.4. Bioactivity-Guided Fractionation

Purification of DSP-like compound(s) from Maine shellfish was achieved through bioactivity guided fractionation following a modification of the method described in [39]. First, a homogenized composite sample of 12 mussels (*Mytilus edulis*), collected in 2016 from the Gulf of Maine during the *D. norvegica* bloom and previously shown to contain >0.35 ppm OA equivalent activity using the PPIA kit and 0.67 ppm OA eq. using the MaxSignal quantitative ELISA, both described in the “Commercial Test Kit” section above, was extracted following the standard procedure used in the LC-MS/MS analysis described below (2 g homogenate extracted twice with 9 mL each of MeOH with the final volume adjusted with MeOH to 20 mL). A 2 mL sub-sample was hydrolyzed with 250 µL of 2.5M NaOH followed by heating in a water bath at 76 °C for 40 min. After cooling to room temperature, the sample was neutralized with 250 µL of 2.5M HCl. A sub-sample of hydrolyzed extract (500 µL) was injected onto an Agilent 1200 Series HPLC system equipped with a G1363A 900 µL extended loop injection kit and a 1260 Series model G1364C fraction collector (Agilent Technologies, Waldbronn Germany). Separations were achieved using a 4.6 × 150 mm, 5 µm particle size, Zorbax Eclipse XDB-C18 column (Agilent Technologies, Waldbronn Germany) at a flow rate of 500 µL/min using the following elution profile: 70% A:30% B hold for 2 min, increasing linearly to 100%B over 20 min, hold at 100%B for 5 min, decrease to 70%A:30% B over 3 min, hold at 70% A:30% B for 5 min. Mobile phase A consisted of 100% water with 0.1% TFA and mobile phase B consisted of 100% acetonitrile with 0.1% TFA following [39]. Fractions were collected every minute over the entire 35 min run time. All fractions were tested for protein phosphatase inhibitory activity using the commercial kit described in Section 4.3. As a positive control, a homogenate of clam (*Mercenaria mercenaria*), previously shown to be <LOD for DSP toxins by LC-MS/MS, was spiked with 0.16 ppm each of methanolic reference solutions of OA, DTX1, and DTX2, then extracted, fractionated, and analyzed for PPIA activity as described above.

### 4.5. Liquid Chromatography-Mass Spectrometry

#### 4.5.1. Lipophilic Toxin Screening by Liquid Chromatography-High Resolution Mass Spectrometry (LC-HRMS) Analysis

Initial lipophilic toxin screening using LC-HRMS analyses, performed at NRC Canada, of the first set of 10 samples collected by ME DMR in 2016 for DSTs as well as azaspiracids, pectenotoxins, and yessotoxins was performed according to [40] and [41].

#### 4.5.2. Q1 Scanning and MS/MS Analysis of the Unknown DST-Like Compound

Initial LC-MS experiments on the semi-purified extract from the 26–27 min fraction obtained from the PPIA bioactivity-guided fractionation of Sample 2 (from Figure 1 and Table 1) were performed using an Acquity Ultra-Performance Liquid Chromatography system (Waters Corporation, Manchester, UK) coupled to a Sciex QTrap 5500 mass spectrometer equipped with a Turbo V ionization source (SCIEX, Framingham, MA, USA). The column used for separations was a Waters BEH C18 (1.7 µm, 1.0 mm × 150 mm) (Waters Corp., Milford, MA). The aqueous mobile phase (A) consisted of 2 mM ammonium formate and 50 mM formic acid in water. The organic mobile phase (B) consisted of 2 mM ammonium formate and 50 mM formic acid in 95% acetonitrile/5% water. For initial method development studies, gradient conditions started at 50% B, were maintained for two min at 50% B, and were then linearly increased to 70% B in four min, followed by 100% B in two min, held at 100% B for five min, then decreased to 50% B in 0.5 min and lastly, were held at 50% B for 4.5 min. The total run time was 18 min at a flow rate of 0.12 mL/min. The column temperature was maintained at 40 °C while the autosampler temperature was 10 °C. The injection volume was 5 µL.

The electrospray ionization (ESI) source parameters were as follows: source temperature 550 °C, ion spray voltage −4500 V, curtain gas 25 psi, gas 1 and 2 both 40 psi. Full scan, negative ionization mode data were collected using a mass range from 500 to 900 Da and a scan rate of 200 Da s^−1^. Product ion scans for *m/z* 819.50 were collected using a mass range from 100 to 900 Da and a scan rate of 1000 Da s^−1^. The declustering and entrance potentials were −110 V and −10 V, respectively, and for product ion scans the collision energy was −70 V and collision cell exit potential was −15 V. Analyst® chromatography software (ver. 1.6.2, SCIEX, Framingham, MA, USA) was used for data visualization and analysis. 

#### 4.5.3. LC-HRMS Analysis of Dihydrodinophysistoxin-1

LC-HRMS measurements of the semi-purified extract from the 26–27 min fraction of Sample 2 (from Figure 1 and Table 1) was performed using a Nexera LC system (Shimadzu, Columbia, MD, USA) coupled with a Q Exactive Hybrid Quadrupole-Orbitrap mass spectrometer (Thermo Scientific, Waltham, MA, USA). The column and mobile phases were the same as described in Section 4.5.2., with the LC run time shortened to 15 min. Gradient conditions starting at 50% B were maintained for two min, then linearly increased to 70% B in four min, followed by 99% B in two min, held at 99% B for two min, then decreased to 50% B in 0.5 min and held at this level for 4.5 min to re-equilibrate. All other chromatography and autosampler settings were the same as described in Section 4.5.2.

Analytes were ionized using ESI in negative mode with source conditions as follows: spray voltage −3000 V, capillary temperature 320 °C, sheath gas 5 arbitrary units (au), and aux gas 0 au. A targeted-single ion monitoring (SIM)/data dependent (dd)-MS^2^ experiment was performed on the sample. The instrument was set to monitor and perform MS/MS on *m/z* 819.49002. The parameters for SIM were a mass resolution setting of 70,000, automatic gain control (AGC) of 2 × 10^5^, maximum injection time of 200 ms, and an isolation window of 2 *m/z*. The parameters for dd-MS^2^ were as follows: mass resolution setting of 35,000, AGC 2 × 10^5^, maximum injection time 100 ms, normalized collision energy 35. The dd settings to initiate MS/MS was a minimum AGC of 8 × 10^3^.

#### 4.5.4. LC-MS/MS Selected Reaction Monitoring (SRM) Analysis for OA, DTX1, DTX2, and Dihydro-DTX1

LC-MS/MS testing by SRM was performed using an Acquity Ultra-Performance Liquid Chromatography system coupled to a Sciex QTrap 5500 mass spectrometer equipped with a Turbo V ionization source. The protocol “LC-MS/MS Method for the Detection of DSP Toxins in Shellfish” [42] that was adopted in 2017 by the Interstate Shellfish Sanitation Conference (ISSC) for use in the US National Shellfish Sanitation Program (NSSP) [30] was followed with minor modifications to include the measurement of dihydro-DTX1 as detailed below. All samples were subjected to alkaline hydrolysis, following the referenced protocol, to measure total (free plus esterified) toxins. The column, mobile phase, gradient conditions, and ESI source parameters were the same as those used for LC-HRMS measurements (Section 4.5.2) and in the ISSC protocol. Data acquisition was in negative ionization mode using SRM. The SRM parameters for dihydro-DTX1 were as follows: Q1 *m/z* 819.5, Q3 *m/z* 255.2 and 151.1, dwell time 100 ms, declustering potential -110 V, entrance potential −10 V, collision energy −70 V, and collision cell exit potential -15 V. The peak area of the SRM transition *m/z* 819.5→255.2 was used for quantitation, while the *m/z* 819.5→151.1 SRM transition was used for confirmation. In the absence of a standard, quantitation of dihydro-DTX1 was performed using the calibration curve of DTX1. Analyst® chromatography software (ver. 1.6.2, SCIEX, Framingham, MA, USA) was used for peak area integration and quantitation. 

### 4.6. Analysis of Gulf of Maine Shellfish for Dihydro-DTX1 by LC-MS/MS SRM and Comparison with PPIA

To compare the determination of dihydro-DTX1 by LC-MS/MS SRM (as described in Section 4.5.4) to PPIA (as described in Section 4.3), 48 shellfish samples collected by ME DMR during blooms of *D. norvegica* in 2016 and 2018, each comprising of ≥12 composited individuals and representing mussels (*Mytilus edulis*), oysters (*Crassostrea virginica*), and clams (*Spisula solidissima* and *Mya arenaria*), were analyzed using both methods. For any samples found to be greater than the working range of the PPIA kit (>0.35 ppm OA eq.), samples were diluted using the kit-provided dilution buffer and re-analyzed. All samples >LOD for the PPIA kit (0.063 ppm) (N = 42) were compared to results determined by LC-MS/MS SRM, quantified using an external DTX1 standard curve, using both linear regression and correlation analysis with the program GraphPad Prism (ver. 5.01 for Windows, GraphPad Prism Software, San Diego, CA, USA).

During the 2018 *D. norvegica* bloom in the Gulf of Maine, three species of shellfish: mussels (*Mytilus edulis*), clams (*Spisula solidissima*), and oysters (*Crassostrea virginica*), were collected approximately weekly (≥12 composited individuals per sample) between May 30th and June 18th from a single location (Blue Hill Falls, Figure 1) and analyzed by LC-MS/MS SRM (as described in Section 4.5.4) to look for species-specific differences in the accumulation of dihydro-DTX1. 

### 4.7. Testing of a Gulf of Maine Dinophysis norvegica Culture for DST Production

Two new cultured clonal isolates of *D. norvegica* (DNBH-FB4 and DNBH-B3F) were established in culture in May 2018 from surface water collected from Blue Hill Falls, Maine, following the single-cell isolation methods described by [43]. At the time of water collection, the salinity was 30 psu and the water temperature was 12 °C. During isolation and maintenance of the culture, *D. norvegica* were fed *Mesodinium rubrum,* which had been previously raised on *Teleaulax amphioxeia* isolated from Japan [44] following the protocols of [45] as modified by [46]. The dinoflagellate, ciliate, and cryptophyte cultures were grown in modified f/6-Si medium [47] and a salinity of 30 at 15 °C in dim light (40 µmol photons·m-2·sec-1) under a 14 h:10 h light:dark photocycle. 

To assess the toxigenicity of DNBH-FB4 and DNBH-B3F, cells were inoculated into fresh medium, salinity 30 psu, at 400 cells·mL^−1^, fed *M. rubrum* at a 1:10 ratio of prey to predator, and monitored every 3 days for the complete consumption of *M. rubrum* by examining 1 mL subsamples in a Sedgewick-Rafter counting cell at 100x using an Olympus CX31 light microscope (Olympus America, Waltham, MA, USA). Three days after all ciliate prey were consumed, (i.e., during late exponential growth of the dinoflagellate) the culture was harvested for toxin analysis.

The harvested cultures were gently separated into cells (intracellular toxins) and medium (extracellular toxins) using a 10 µm sieve and the components were extracted and analyzed for toxins separately. Cells and medium were bath-sonicated at room temperature for 15 min (Branson 5800 Ultrasonic Cleaner, 5800) and loaded onto an Oasis HLB 60 mg cartridge (Waters Corporation, Millford, MA, USA) that was previously equilibrated with 3 mL of MeOH and 3 mL of GenPure water. The cartridge was then washed with 6 mL of GenPure water, blown dry, and eluted with 1 mL of 100% MeOH into a glass 1.5 mL high recovery LC vial and stored at −20 °C until analyzed. A portion of the sample underwent alkaline hydrolysis to enable the quantitation of total DSP toxins (free plus esterified) following [48]. Extracts, original and alkaline hydrolyzed, were analyzed using an Acquity liquid chromatography system coupled with a Xevo mass spectrometer with electrospray ionization (Waters, Milford, MA, USA) following the DSP and PTX2 analytical methods described by [49]. 

Dihydro-DTX1 was detected using selected reaction monitoring (SRM) in negative ion mode with the transitions *m/z* 819.5→255.1, 819.5→819.5, 819.5→151.1, and 819.5→113.1. Quantitation was performed using the former SRM transition; concentrations were calculated using an external DTX1 standard curve with MassLynx 4.1 software (Waters Corporation, Millford, MA, USA). Matrix spikes, final concentration of 12.5 ppb DTX1, were conducted to confirm separation from DTX1. Toxin data are presented as toxin concentration per mL of culture, and free vs. esterified, with the latter being calculated through the subtraction of free toxins from total toxins.

## Figures and Tables

**Table 1:** Results from the analysis for mussel (*Mytilus edulis*) samples collected during the 2016 *Dinophysis norvegica* bloom in the Gulf of Maine. (PPIA) protein phosphatase inhibition assay (Okadaic Acid (DSP) PP2A kit, Abraxis Inc., USA), (ELISA) enzyme-linked immuno-sorbent assay (qualitative-NEOGEN Reveal 2.0 for DSP, quantitative-Bioo Scientific MaxSignal Okadaic Acid (DSP)), (LC-MS/MS) liquid chromatography-tandem mass spectrometry performed (1) according to the selected reaction monitoring (SRM) method approved by the National Shellfish Sanitation Program for analyzing clams for OA, DTX1, and DTX2 and (2) with the additional transitions for dihydro-DTX1. All units are in ppm.

Sample ^a^	PPIA	ELISA		LC-MS/MS	
		Qualitative	Quantitative	OA, DTX1, DTX2	Dihydro-DTX1 ^b^
**1**	0.20	Positive	0.24	ND	0.24
**2**	>0.35	Positive	0.67	ND	1.08
**3**	0.08	Negative	0.08	ND	0.06
**4**	0.10	Negative	0.07	ND	0.07
**5**	0.28	Positive	0.34	ND	0.79
**6**	0.17	Positive	0.16	ND	0.20
**7**	0.19	Negative	0.14	ND	0.21
**8**	0.14	Negative	0.15	ND	0.16
**9**	<0.06	Negative	<LOD	ND	ND
**10**	<0.06	Negative	0.06	0.04 DTX1	Trace

^a^ Numbers correspond to sampling locations shown in Figure 1: (1) Stinson Neck Causeway, (2) Oak Point, (3) Pretty Marsh Harbor, (4) Trenton Sea Plane Ramp, (5) Googins Ledge, (6,7) Bar Harbor, (8) Raccoon Cove, (9,10) Lumbo’s Hole (control site outside of bloom area). Sampling site coordinates provided in Table S1. ^b^ (M-H)^−^ *m/z* 819.5 fragmenting to *m/z* 255.2 (for quantification) and 151.1 (for confirmation), quantified using an external DTX1 standard. ND: Not detected. Trace: >LOD 0.2 ppb and <LOQ 0.6 ppb.

**Table 2:** Concentrations of dihydro-DTX1 and PTX2 in methanolic extracts from the intracellular and extracellular fractions of two cultures of *D. norvegica* isolated from the Gulf of Maine in 2018, as measured by LC-MS/MS. OA, DTX1, and DTX2 were also analyzed, but were not detected.

Isolate	dihydro-DTX1 (ppb)						PTX2 (ppb)	
	Intracellular			Extracellular			Intracellular	Extracellular
	Free	Esterified	Total	Free	Esterified	Total		
*DNBH-FB4*	1	60.6	61.5	1.3	5.3	6.5	36.3	3.9
*DNBH-B3F*	0.5	43.1	43.6	1.4	7.1	8.5	26.5	2.5

## Supplementary Materials

The following are available online at https://www.mdpi.com/2072-6651/12/9/533/s1, Table S1: Sampling site coordinates for shellfish and phytoplankton samples collected during the 2016 *Dinophysis norvegica* bloom depicted in Figures 1 and 2. Figure S1: MS/MS spectrum of *m/z* 819.5 peak detected in the 26–27 min fraction from the bioactivity guided fractionation extract of mussel (*Mytilus edulis*) collected during 2016 *Dinophysis norvegica* bloom in the central coast of the Gulf of Maine, USA; Figure S2: Structure of precursor ions and proposed product ion structures for okadaic acid (OA), dinophysistoxin 1 (DTX1), and dinophysistoxin 2 (DTX2).
