# Supplementary material for: Dihydrodinophysistoxin-1 Produced by Dinophysis norvegica in the Gulf of Maine, USA and Its Accumulation in Shellfish
Source: Toxins (Basel). 2020 Aug 20;12(9):533. doi: 10.3390/toxins12090533 (PMC7551465; doi:10.3390/toxins12090533)
Supplement: Supplementary file 1 [file toxins-12-00533-s001.pdf]

Supplementary Materials: Dihydrodinophysistoxin-1 Produced by *Dinophysis norvegica* in the Gulf of Maine, USA and Its Accumulation in Shellfish

Jonathan R. Deeds, Whitney L. Stutts, Mary Dawn Celiz, Jill MacLeod, Amy E. Hamilton, Bryant J. Lewis, David W. Miller, Kohl Kanwit, Juliette L. Smith, David M. Kulis, Pearse McCarron, Carlton D. Rauschenberg, Craig A. Burnell, Stephen D. Archer, Jerry Borchert and Shelley K. Lankford

Table S1. Sampling site coordinates for shellfish and phytoplankton samples collected during the 2016 *Dinophysis norvegica* bloom depicted in Figures 1 and 2.

	Map Number	Sampling Site	Latitude	Longitude
**Figure 1**	1	Stinson Neck Causeway	44.21074	-68.59489
	2	Oak Point	44.44718	-68.3323
	3	Pretty March Harbor	44.33965	-68.41552
	4	Trenton Sea Plane Ramp	44.442443	-68.35756
	5	Googins Ledge	44.447898	-68.268982
	6	Bar Harbor	44.39342	-68.20833
	7	Bar Harbor	44.39342	-68.20833
	8	Raccoon Cove	44.47093	-68.27944
	9	Lumbos Hole	43.79553	-69.94557
	10	Lumbos Hole	43.79553	-69.94557
**Figure 1&2**		Lincolnville	44.28237	-69.0074
		Searsport	44.46035	-68.8829
		Dice Head	44.38107	-68.8197
		Eggemoggin Reach	44.2908	-68.6926
		Blue Hill Falls	44.37713	-68.5612
		Flye Point	44.25945	-68.5195
		Lamoine State Park	44.45125	-68.2999
		Salsbury Cove	44.43248	-68.2852
		Waukeag	44.47156	-68.184

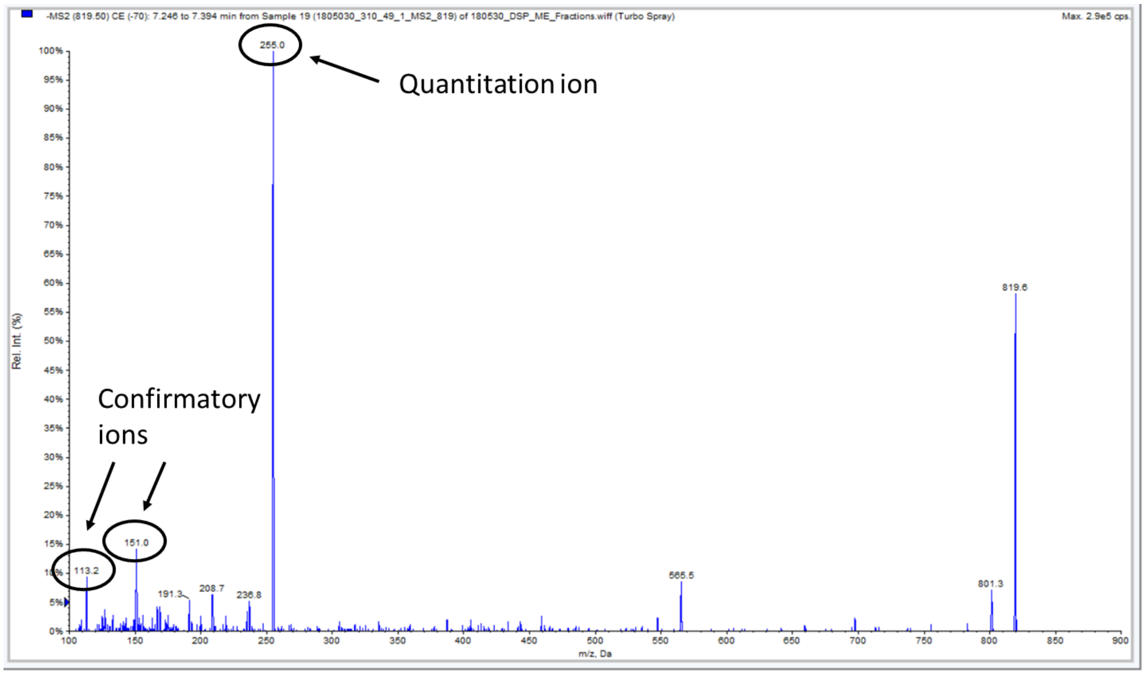

Figure S1. MS/MS spectrum of *m/z* 819.5 peak detected in the 26–27 min fraction from the bioactivity guided fractionation extract of mussel (*Mytilus edulis*) collected during 2016 *Dinophysis norvegica* bloom in the central coast of the Gulf of Maine, USA.

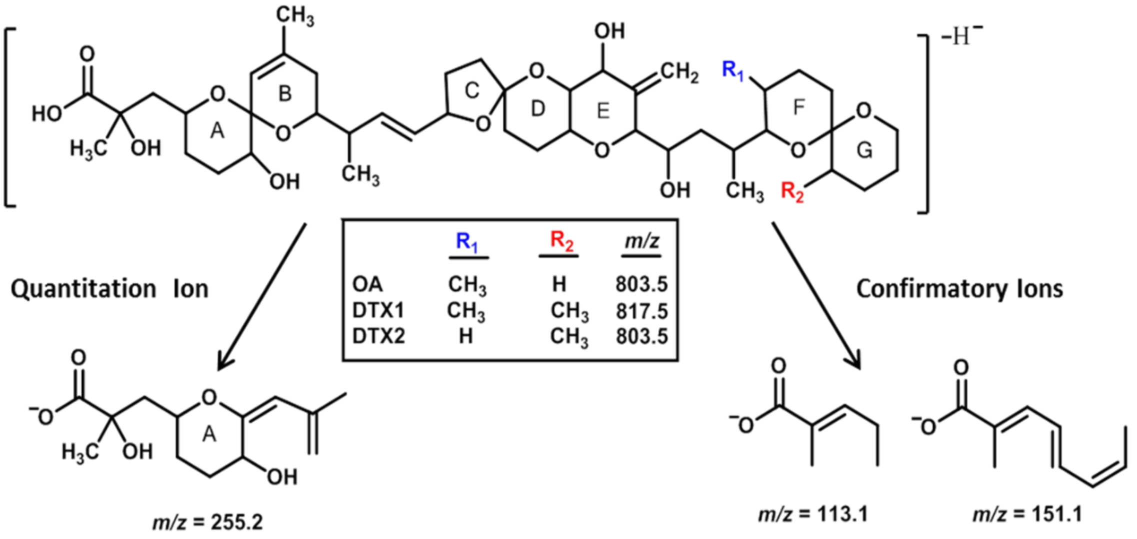

Figure S2. Structure of precursor ions and proposed product ion structures for okadaic acid (OA), dinophysistoxin 1 (DTX1), and dinophysistoxin 2 (DTX2).
